# The Value of Extract of Malt and Loefland’s Concentrated Liebig’s Food for Infants, and an Account of Their Introduction to the Medical Profession

**Published:** 1878-08-20

**Authors:** W. A. Greene

**Affiliations:** Macon, Ga.


### SOUTHERN MEDICAL RECORD.

VOL. VIII. ATLANTA, GA., AUGUST 20, 1878. NUMBER 8.

EDITORS:

THOMAS S. POWELL, M. D. W. T. GOLDSMITH, M. D. R. C. WORD, M. D.

*R. C. WORD, M. D., Managing Editor.*

SUBSCRIPTION—$2.00 PER ANNUM, IN ADVANCE.

☞ All Communications and Letters on Business connected with THE RECORD, for 1877 and 1878, must be addressed to the Managing Editor.

### *ORIGINAL AND SELECTED.*

### THE VALUE OF EXTRACT OF MALT AND LOEFLAND'S CONCENTRATED LIEBIG'S FOOD FOR INFANTS, AND AN ACCOUNT OF THEIR INTRODUCTION TO THE MEDICAL PROFESSION.

BY W. A. GREENE, M.D., Macon, Ga.

Liebig's Extract of Malt and Food for Infants have now been long enough before the world to enable most men to form their own opinions as to their specific value as articles of food and articles of medicine. Nevertheless, it may not be amiss, on our part, to point out briefly what has been ascertained as to their worth, and to indicate some of the many uses to which they ought to be put.

Meat, as an article of diet, owes its value partly to the mineral substances it contains, and partly to the organic compounds, albuminoid and oleaginous. The mineral substances probably undergo little change in the human body; the oleaginous do not now concern us; the albuminoid, having been ingested, are in the stomach reduced to a uniform substance, termed albuminose or peptone, by means of the acid and pepsin of the gastric secretion. All albuminoid substances are so—including albumen, fibrin, etc.—but the conversion of amylaceous principles into saccharine, in the human economy, is accomplished by the secretions from the salivary glands and pancreas, which secrete analogous principles—that existing in the salivary secretions, known as ptyalin, and that in the pancreatic juice as pancreatin. Now, there is in malted barley a substance analogous to these, which has a strong power of changing starch into sugar, as the pepsin in the gastric juice has the power of converting albuminous substances into peptone. This substance in malted barley is called *diastase*, and is formed during the process of germination or malting, a very small proportion of which converts an almost indefinite proportion of starch into sugar. Now, when the animal system is incapable, from abnormal causes, of converting starch food into sugar, we must aid it by supplying an artificial saliva, as it were, thus making extract of malt a most valuable therapeutic agent for the treatment of enfeebled and exhausted constitutions, being rich in both muscle and fat, producing material, the diastase possessing the property of converting about two thousand times its weight of starch into glucose, thus preparing a product which normally enters the blood and at once becomes a nutritious element. We at once perceive how valuable must this remedy be in dyspeptic and all other stomachic disorders, caused by non-assimilation of starch food. There are varied combinations of other medicines with extract of malt which infinitely increases and qualifies its good effects, which I shall reserve for another paper.

The careful selection of food for infants who are deprived of the mother's milk is a subject of more importance than generally supposed. If it was properly comprehended, the common error of recommending arrow-root, corn starch, tapioca, rice, oatmeal, or other starch food, would not occur. It is a grave error, and one very injurious to infants, for this reason: before teething, animal diastase, the starch-converting substance, not being secreted, all such food, when taken into the stomach, remains undigested, not as food, but a jelly-like mass, non-assimilable, foreign, and sure to irritate the delicate membranes of the alimentary canal, producing chronic diarrhœa and "summer complaint," which annually destroys its thousands of infants. But after teething, a substance is secreted by the salivary glands, which has the power of changing starch-food and converting it into malt-sugar. Of course, before the teeth are formed, there is no saliva—no secretion from the salivary glands; consequently, no animal diastase. But in malted barley, there is a substance, called vegetable diastase, precisely similar to that formed in the salivary secretions after teething. It is well known that all farrinacious food is very valuable as nutriment; but who, with this knowledge of facts, would think of giving it to infants as an article of diet? Yet, it is done every day—and why? Because these physiological facts are either not known or are neglected. Long before the French chemist, Miache, read his *Memoire* before the French Academy, announcing his discovery of diastase in saliva, and its functions, and before Baron Justus von Liebig discovered vegetable diastase in malted barley, had our mothers and nurses found that "chewed biscuit" was "good for the babie." Then knowledge was practical and experimental; but the celebrated chemist demonstrated it scientifically, from which resulted the noted formula for preparing "Liebig's Soup for Infants," which proved such a blessing to the human race, and saved myriads of lives.

In 1865, Mr. Edward Loefland established a factory at Stuttgart, Germany, solely for the manufacture of this "soup" in the form of a concentrated extract. Professor Liebig had published his formula for the infants' soup in 1863, which was composed of wheat flour, malt flour, and potana in certain proportions, which, when properly prepared, according to directions, was chemically identical with the mother's milk. Although scientifically so correct, it proved a failure, practically, from the tedious and inconvenient process of its preparation. This great, good, and learned man was humiliated and mortified by this lack of appreciation of his efforts by his countrywomen. They never tired in the appropriation of his discoveries which would adorn and beautify their persons, regardless of expense, time or fatigue, but the babie-food was too much trouble to prepare!

In order both to illustrate the duties and sacrifices which should characterize maternal devotion, and to prove the absolute correctness of his investigations, with his own hands he prepared the "soup" for a delicate grandchild, who required to be thus nourished, several times each day. The result was as gratifying as his example was brilliant.

About this time, the practical and zealous Loefland came to the assistance of Professor Liebig, and suggested that if the above obstacles could be overcome, and the "soup," in its original composition, brought to the notice of the profession and the public in an easily and conveniently prepared form, its great benefits would not be lost, but would preserve its identity and perpetuate its existence in the upward and onward mission for which it was instituted and sent forth to accomplish.

In the spring of 1866, Professor Liebig visited the establishment of Loefland, in company with Professor von Fehling, professor of chemistry at the Polytechnic School of Stuttgart. The former expressed his entire satisfaction, not only of the product obtained, but also approved the theory, apparatus, and entire management of the establishment. Thus was perfected "Loefland's Concentrated Liebig's Food for Infants."

While these scientists were inspecting this establishment, Professor Liebig informed Mr. Loefland that he had prepared, at his Laboratory in Munich, a malt-sugar, according to the old pharmacopæia, and that he would suggest as a nutriment, resolvent, and emollient, the manufacture of a concentrated extract of malt that combined all the nitrogenous, albuminous bodies, the phosphates, and a large proportion of malt-sugar. Mr. Loefland had all the facilities for making such an extract in his establishment. Professor Liebig urged him to go ahead. Loefland again began to experiment and manufacture—the products of which experience were exhibited at the Exposition, in Paris, in 1867, and secured the first prize. This was its first triumph before the public and the profession, since which time it has been the recipient of numerous awards wherever presented, and has won the confidence of all who have used it, in consequence of which the demand increased to such an extent that the old arrangement was too limited, and the space has been greatly enlarged, and improved machinery procured, until to-day it is, indeed, a mammoth concern, supplying pure, concentrated, and reliable malt to all portions of the globe. I do not hesitate to recommend their preparations to the profession and public as unexcelled in all respects, and invaluable for the purposes indicated.

In another paper, I will speak more particularly of its merits, and also refer to some of its valuable combinations.

I should have stated that Mr. A. C. Dung, 61 Bowery, New York, and John Wyeth & Brother, 1428 Walnut street, Philadelphia, are the exclusive agents for its sale in the United States. But it will soon be found in every drug store in the country.

